# Enhancer in cancer pathogenesis and treatment

**DOI:** 10.1590/1678-4685-GMB-2022-0313

**Published:** 2023-08-04

**Authors:** Zhuo Sun, Jinbo Fan, Yixiong Dang, Yufeng Zhao

**Affiliations:** 1 Xi’an Medical University, Xi’an Key Laboratory of Pathogenic Microorganism and Tumor Immunity, Weiyang District, Xi’an, Shaanxi, China.; 2 Institute of Basic Medical Sciences, No.1 XinWang Rd, Weiyang District, Shaanxi, China.; 3 Xi’an Medical University, School of Public Health, Weiyang District, Xi’an, 710021 Shaanxi, China.

**Keywords:** Enhancer, super-enhancer, enhancer reprogramming, cancer, BETi

## Abstract

Enhancers are essential *cis*-acting regulatory elements that determine cell identity and tumor progression. Enhancer function is dependent on the physical interaction between the enhancer and its target promoter inside its local chromatin environment. Enhancer reprogramming is an important mechanism in cancer pathogenesis and can be driven by both *cis* and *trans* factors. Super enhancers are acquired at oncogenes in numerous cancer types and represent potential targets for cancer treatment. BET and CDK inhibitors act through mechanisms of enhancer function and have shown promising results in therapy for various types of cancer. Genome editing is another way to reprogram enhancers in cancer treatment. The relationship between enhancers and cancer has been revised by several authors in the past few years, which mainly focuses on the mechanisms by which enhancers can impact cancer. Here, we emphasize SE’s role in cancer pathogenesis and the new therapies involving epigenetic regulators (BETi and CDKi). We suggest that understanding mechanisms of activity would aid clinical success for these anti-cancer agents.

## Characteristics and types of enhancers

### Characteristics of enhancers

Enhancers are orientation-independent *cis*-acting regulatory elements that increase transcription activity from a distant promoter. Enhancer regions have higher DNA accessibility and nucleosomes in enhancer regions have signature histone modifications such as H3K4me1 and H3K4me2 and are usually depleted of H3K4me3 (Kundaje et al., 2015). There are four enhancer-activation states: inactive, primed, poised and active. Inactive enhancers are buried in compact heterochromatin and have no transcription factor association. Primed enhancers are bound by transcription factors and are inside Dnase I hypersensitive open chromatin, but still need further signal or cofactors binding to exert active enhancer function. Poised enhancers are mostly found in embryonic stem cells and are primed enhancers with repressive histone modifications, such as H3K27me3 (Rada-Iglesias et al., 2011). Active enhancers are marked by H3K27Ac. They are actively transcribed into enhancer RNA (eRNA) by RNA polymerase II and function to boost target gene expression.

### Types of enhancers

There are mainly two types of enhancers depending on their activation stimuli and function: cell type-specific enhancers and signal-dependent enhancers (also called inducible enhancers). Cell type-specific enhancers represent a large proportion of all enhancers. In a recent study, researchers identified active enhancers across 10 human tissues, and most of them are tissue-specific enhancers (Xiong et al., 2018). Enhancer-target networks and enhancer RNA profiles are robust identifying features for different cell and tissue lineages(Cao et al., 2017; Tu et al., 2021). All different cell types in the human body contain the same genome, and one of the vital factors that determines cell type-specific gene expression is cell type-specific enhancers. Although mammalian genomes contain millions of potential enhancers, only a small percentage is active in any given cell type. For a specific gene locus such as T-cell acute lymphocytic leukaemia 1 (*TAL1*), several developmental enhancers have been identified and different choices and combinations of these enhancers are used for different cell types (Heinz et al., 2015). The -3.8kb (upstream) and +19kb (downstream) enhancers drive *TAL1* expression in human umbilical vein endothelial cells and hematopoietic stem and progenitor cells (Sánchez et al., 1999; Göttgens et al., 2004), and the +51kb enhancer is essential for *TAL1* transcription in K562 erythroid cells (Delabesse et al., 2005). These enhancers are activated according to the cell’s specific developmental stage and environmental stimuli and work to boost the expression of cell identity genes. 

Pioneer TFs, lineage-dependent TFs (LDTFs), and signal-dependent TFs (SDTFs) work collaboratively to select and activate inactive and poised enhancers and establish lineage-specific gene expression (Heinz et al., 2010). Chromatin remodelers and histone modifiers are also important players in the activation of cell-type specific enhancers (Park et al., 2021). There are two types of mechanisms by which LDTFs and SDTFs work together to select and activate cell type-specific enhancers (Heinz *et al.*, 2015). For one mechanism, there is a hierarchical relationship between LDTFs and SDTFs binding, where LDTFs act as pioneer factors that initially select enhancers and the binding of SDTFs can further induce the enhancer activity. For the other mechanism, SDTFs contribute directly to enhancer selection through collaborative interactions with LDTFs.

While cell type-specific enhancers play a vital part in cell-type determination, some enhancers serve as main regulators of gene expression in response to various acute signaling pathways, where signal-dependent transcription factors preferentially bind to enhancers (Tan et al., 2023). These enhancers belong to signal-dependent enhancers. Examples of signal-dependent enhancers include hormone-responsive enhancers (Shlyueva et al., 2014; Hoffman et al., 2022), virus-inducible enhancers (Thanos et al., 1993, 1996), metal-responsive enhancers (Karin et al., 1987; Westin and Schaffner, 1988), and NF-kappa B and cytokine-inducible enhancers (Collins et al., 1995). 

### Super-enhancer

Super-enhancer (SE) is the term used to describe clusters of active enhancers that are in a high density in a genomic region. Super-enhancers have the function of regulating genes essential for cell identity determination. SEs are enriched with more TFs, Mediator complexes, and RNA Pol II molecules than typical enhancers, which results in higher transcriptional activity (Yoshino and Suzuki, 2022). BRD4, p300, CDK7, CDK9, and MED1 (Mediator Complex Subunit 1) are all important factors that characterize SEs (Khan and Zhang, 2019). High concentrations of transcription factors, co-activators (BRD4 and p300), and RNA polymerase II forms transcriptional condensates to drive the interaction between promoter and enhancer. SE has been implicated in the pathogenesis of various types of cancer. SEs are extremely sensitive to perturbations by drugs (Bradner et al., 2017). A small change in concentrations of SE components causes drastic changes in SE-associated gene expression (Lovén et al., 2013). This has been utilized when exploring potential therapy to treat cancer and will be discussed in detail in part 4.1 targeting mediators of super-enhancer function.

## Molecular mechanism of enhancer function

### Polymerase II and eRNA

Polymerase II is recruited to active enhancers and produces short transcripts. Pol II is then transferred from enhancers to promoters to initiate transcription at the target gene (Gibbons et al., 2022). SEs are characterized by abundant association with Pol II and are most sensitive to interference with Pol II function. Inhibition of Pol II function through CDK7 could be utilized in cancer therapy and is discussed in detail in part 4.1.2 CDK7 inhibitors.

The transcripts that come from the transcription of enhancers are called enhancer RNA (eRNA). Most eRNAs are short transcripts (around 500bp) that are non-polyadenylated and unspliced (Andersson et al., 2014a,b). Only a small number of eRNAs are long (several kb in size) that are polyadenylated (Koch et al., 2011). eRNA production is predictive of active enhancer function (Melgar et al., 2011; Andersson *et al.*, 2014a; Core et al., 2014; Henriques et al., 2018) and eRNA level correlates with the transcriptional activity of their target gene (Kim et al., 2010). Transcription from enhancers can be unidirectional (Koch *et al.*, 2011) but is mostly bidirectional (Kim *et al.*, 2010). eRNA is typically unstable (Lubas et al., 2015), so it is not always detectable even when the enhancer is functional (Andersson *et al.*, 2014a; Mikhaylichenko et al., 2018). There has been evidence that eRNA might be contributing to enhancer function through several mechanisms, including increasing chromatin accessibility (Mousavi et al., 2013), recruitment of cofactors (Kaikkonen et al., 2013; Bose et al., 2017), maintenance of transcription factor binding (Sigova et al., 2015), enhancer-promoter contact (Li et al., 2013), and phase separation (Nair et al., 2019). 

### Promoter-enhancer interaction

The function of an active enhancer is dependent on the physical interaction between the enhancer and its target promoter. Several models have been proposed for enhancer-promoter communication, including tracking, chaining, and looping (the loop extrusion model) (Furlong and Levine, 2018). In the tracking model, Pol II binds to an enhancer through interaction with transcription factors and tracks along the chromatin, pulling the enhancer with it until it reaches its target promoter. In the chaining model, TFs bound to the enhancer oligomerize and form a chain to interact with the target promoter. In principle, the tracking and chaining model could only work in short-range interactions, and the most widely accepted model of action is the loop extrusion model. The loop extrusion model incorporates looping and tracking. In the loop extrusion model, cohesin complexes form tripartite rings around chromatin and translocate along the chromatin fiber in opposite directions, therefore actively extruding a progressively larger chromatin loop until they are stopped by CTCF boundary elements (Fudenberg et al., 2016). The chromatin loop, formed between the enhancer and its target promoter, is called an enhancer-promoter loop. The enhancer-promoter loop provides the structural basis for enhancer function. There are many cofactors that are involved in the enhancer-promoter loop, such as CTCF, cohesin, BRD4, the Mediator complex, RNA Polymerase II, chromatin modifiers, transcription factors, pioneer factors, and transcription coactivators. It is important to note that some evidence shows that some regulatory elements might have both enhancer and promoter functions, and transcription initiation and transcriptional enhancement may not be mutually exclusive functions for a specific regulatory element (Andersson and Sandelin, 2020). 

### TAD

Since enhancers can be as much as 1Mb away from their interacting promoters (Furlong and Levine, 2018), their interaction is based on the 3D organization of the genome (Robson et al., 2019; van Steensel and Furlong, 2019). Enhancers work in the context of chromatin domains and preferentially interact with promoters that are in the same topological associating domains (TAD) rather than a nearby TAD (Symmons et al., 2014). Disruption of TADs could cause improper enhancer-promoter interactions that result in pathogenic phenotypes (Lupiáñez et al., 2015).

### Phase separation

Phase separation has been recently discovered to be an important part of enhancer function. Phase separation is the formation of membraneless organelles inside the cell when groups of molecules interact with each other. Phase separation plays an important part in enhancer function and gene regulation (Sabari et al., 2018; Nair et al., 2019; Zhang et al., 2021; Lee et al., 2022). On the other hand, it is also shown to be essential in decommissioning of enhancers (Jia et al., 2021). It is also discovered to be an important mechanism of aberrant chromatin looping and cancer development (Ahn et al., 2021; Owen et al., 2021; Kabra and Bushweller, 2022; Suzuki and Onimaru, 2022). 

## Function of enhancer in cancer

### Enhancer reprogramming in cancer

There is extensive enhancer reprogramming resulting in the expression of essential players in cancer invasive progression in various types of cancer (Roe et al., 2017; Teng et al., 2020; Yi et al., 2020; Zhou et al., 2020; Ye et al., 2021; Huang et al., 2022). Some enhancers gained activity and drive the expression of oncogenes, while others lose their enhancer activity, which may result in the repression of tumor suppressor genes. 

*cis-acting factors that drive oncogenic enhancer reprogramming*

**Figure 1:**
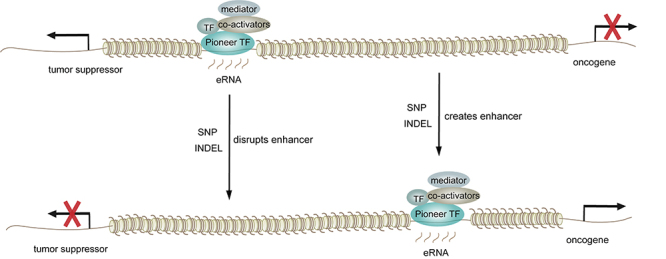
SNP and INDELs can disrupt TF binding motifs in existing enhancers or create new binding motifs for new enhancers, which results in oncogenic gene expression program.

**Figure 2:**
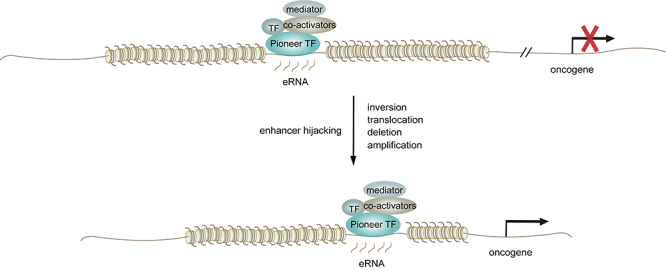
Enhancer hijacking resulting from chromosomal rearrangements can also lead to oncogenic gene expression.

Both *cis*-elements and *trans*-acting factors can induce enhancer reprogramming in cancer progression. *Cis*-acting alterations that drive oncogenic enhancer activity include single-nucleotide polymorphisms (SNPs), small insertions or deletions (INDELs), and enhancer hijacking. SNPs and INDELs represent hereditary cancer predisposition, whereas enhancer hijacking is done through somatic chromosomal rearrangements. SNPs and INDELS result in the gain or loss of enhancer function by creating new or disrupting existing TF binding sites (Figure 1). Enhancer hijacking is the utilization of otherwise harmless enhancers to drive oncogene expression. Large chromosomal rearrangements, including deletions, translocations, inversions, and copy number changes, are responsible for enhancer hijacking (Figure 2).

Large amounts of SNPs linked to diseases have been found to be in noncoding regions and the majority of these SNPs are located in enhancer regions (Hindorff et al., 2009; Maurano et al., 2012; Hnisz et al., 2013; Weinhold et al., 2014; Nasser et al., 2021). The SNP rs2168101 within the SE of the neuroblastoma oncogene *LMO1* influences neuroblastoma susceptibility through differential GATA TF binding and regulation of *LMO1* expression (Oldridge et al., 2015). INDELs acquired upstream of the *TAL1* oncogene introduce de novo binding motifs for the TF MYB, which creates a SE and drives *TAL1* overexpression in primary patient T-cell acute lymphoblastic leukemia (T-ALL) (Mansour et al., 2014). 

Chromosomal translocation causing the repositioning of a single enhancer could result in aberrant expression of oncogene *EVI1* and acute myeloid leukemia (Gröschel et al., 2014). Structural variants that juxtapose *GFI1 (Growth Factor Independent 1)* family oncogenes proximal to active enhancers are discovered to instigate oncogenic activities in medulloblastoma (Northcott et al., 2014). Duplication of an enhancer region near the androgen receptor (AR) locus has been found in advanced prostate cancer that causes therapeutic resistance (Takeda et al., 2018). The duplication causes enhanced AR expression, which undermines the effectiveness of clinical treatment targeting the AR signaling pathway.

*trans-acting factors that drive oncogenic enhancer reprogramming*

Besides *cis*-elements that define the intrinsic ability of an enhancer region to attract TF binding, another important factor is the chromatin landscape, which determines whether the DNA of a robust enhancer is accessible for TF to bind to initiate gene expression. This important aspect of oncogenic enhancer reprogramming involves epigenetic modifications of the enhancers. A myriad of *trans*-acting factors play essential roles in enhancer epigenetic modification, such as chromatin remodelers, epigenetic modifiers, and pioneer TFs.

Chromatin remodelers maintain or change chromatin landscape and DNA accessibility by moving or ejecting nucleosomes. Mutations in the SWI/SNF family of chromatin remodeler account for about 20% of all human cancers (St Pierre and Kadoch, 2017). The ARID1A subunit targets SWI/SNF complex to enhancers and loss of ARID1A impairs the enhancer-mediated transcriptional program of colonic epithelium and drives colon cancer in mice (Mathur et al., 2017). Besides chromatin remodelers that move nucleosomes around, the type of histone variant used in nucleosomes is also an important regulator of enhancer activity in cancer cells. In breast cancer cell lines, H2A.Z occupancy is linked to enhancer activation (Brunelle et al., 2015). Other epigenetic modifiers, such as DNA methyltransferases (Lu et al., 2016; Yang et al., 2016), histone methyltransferases, and demethylases (Sze and Shilatifard, 2016; Andricovich et al., 2018; Tran et al., 2020) are also implicated in oncogenic enhancer function through modulating local active/repressive DNA and histone modifications. 

Pioneer TFs drive chromatin remodeling and opening in enhancer regions and facilitate gene activation. It is observed that metastatic transition in pancreatic ductal adenocarcinoma is accompanied by large-scale enhancer reprogramming. The pioneer TF FOXA1 is a driver of enhancer activation in this process, which leads to a retrograde developmental transition to embryonic foregut endoderm and a more metastatic nature in *vivo* (Roe et al., 2017).

It is important to note that components of the enhancer-promoter loop, whose formation is an essential step in transcription initiation, are also essential *trans*-acting factors in oncogenic enhancer reprogramming. These structural components include CTCF (Fiorito et al., 2016), cohesin (Rao et al., 2017), BRD4 (Lovén et al., 2013), and Mediator (Lovén *et al.*, 2013). BRD4 turns out to be a promising therapeutic target for cancer treatment, which will be discussed in more detail later in this review.

### SE & cancer

Disease-associated SNPs are most frequently found in noncoding regions of the genome and the majority of those noncoding SNPs are located inside enhancers (Hnisz et al., 2013). SEs have been implicated in various types of cancer such as adenoid cystic carcinoma (Drier et al., 2016), basal-like breast cancer (Chen et al., 2019), colon cancer (Göndör, 2020), colorectal cancer (Li et al., 2021; Yu et al., 2021), endometrial carcinoma (Zhang et al., 2016), follicular lymphoma (Heckman et al., 2002), leukemia (Gröschel et al., 2014; Mansour et al., 2014), lung adenocarcinoma (Zhang *et al.*, 2016), multiple myeloma (Delmore et al., 2011; Alvarez-Benayas et al., 2021; Jia et al., 2022), nasopharyngeal carcinoma (Ke et al., 2017; Cai et al., 2020), neuroblastoma (Oldridge et al., 2015), oesophageal squamous cell carcinoma (Jiang et al., 2017), pancreatic cancer (Kim et al., 2021), pleomorphic adenoma (Afshari et al., 2020), prostate cancer (Takeda et al., 2018; Xiao et al., 2022), primary effusion lymphoma (Wang et al., 2020), and rhabdomyosarcoma (Gryder et al., 2020). Known SEs, their target genes, and relative SE formation mechanisms are summarized in Table 1. SEs are associated with key oncogene expressions in many cancer cells. SEs are found near oncogenes in cancer cells, whereas in their healthy counterparts, these SEs are absent (Tang et al., 2020). Many events could lead to SE formation during tumor pathogenesis, including DNA amplification (Zhang *et al.*, 2016) and translocation (Drier *et al.*, 2016). 

**Table 1-:** Known SEs and their target genes in various cancers.

SE formation mechanism	Target gene	Type of cancer
translocation	MYB	Adenoid cystic carcinoma
translocation	EVI1	Acute myeloid leukemia
N/A	KLF5	Basal-like breast cancer
N/A	MYC	Colon cancer
N/A	IL-20RA,PHF19,TBC1D16	Colorectal cancer
focal amplification	MYC	Endometrial carcinoma
translocation	Bcl-2	Follicular lymphoma
aberrant TF binding	TAL1	Leukemia
focal amplification	MYC	Lung adenocarcinoma
translocation	MYC,CCND2, HJURP	Multiple myeloma
N/A	ΔNP63α,ETV6	Nasopharyngeal carcinoma
SNPs in SE	LMO1	Neuroblastoma
N/A	PAK4,RUNX1,DNAJB1,SREBF2	Oesophageal squamous cell carcinoma
N/A	EVI1	Pancreatic cancer
translocation	PLAG1,HMGA2	Pleomorphic adenoma
focal amplification	AR,FOXA1,MYC	Prostate cancer
N/A	MYC, IRF4,MCL1,CCND2,MDM2	Primay effusion lymphoma
translocation	PAX3-FOXO1	rhabdomyosarcoma

### Enhancer and therapy resistance

Therapy resistance is a major issue in anticancer treatment, and the underlying molecular mechanisms are not completely understood. It is recently discovered that enhancer is also an essential factor in cancer therapy resistance (Bao et al., 2019; Canella et al., 2022). BRD4 downregulation is implicated in SE activity and might constitute a novel mechanism for chemoresistance in mixed-lineage leukemia (Canella *et al.*, 2022). Global enhancer reprogramming changes breast cancer transcriptional programs profoundly to promote cellular plasticity and therapy resistance (Bi et al., 2020). It was shown that oncogenic TFs GATA3 and AP1 regulate enhancers that are lost and gained respectively during treatment resistance acquisition. GATA3 is responsible for luminal lineage-specific gene expression, whereas AP1 regulates cancer invasion-related gene programs. The high-order enhancer machinery mediated by differential TF-TF and TF-enhancer interactions is a mechanism of enhancer reprogramming and therapy resistance (Bi *et al.*, 2020).

## Application of enhancer reprogramming in cancer treatment

### Targeting mediators of super-enhancer function

Since it has been observed in cancer cells that enhancers are driving oncogenic transcriptional programs, enhancers have become a potential pharmacological target for interventions of cancer. 

*BETi*

Recently there has been a lot of research effort to explore possibilities to treat cancer with the inhibition of bromodomain and extraterminal (BET) proteins (Whyte et al., 2013). There are four human BET proteins: BRD2, BRD3, BRD4, and testes-specific BRDT, out of which the most studied is BRD4. BRD4 contains two bromodomains, which can bind acetylated lysine on histone tails and transcription factors (Yang, 2004), and a C-terminal motif which can interact with positive transcription elongation factor b (PTEF-b). By binding to acetylated histones, acetylated transcription factors, and PTEF-b, BRD4 serves as a scaffold for transcription machinery to come together. The interaction between BRD4 and PTEF-b permits transcription initiation and elongation (Itzen et al., 2014). BRD4 is widely distributed along the genome and drives the transcription of many cell-lineage-determining genes in somatic cells and oncogenes in cancer (Lovén et al., 2013; Donati et al., 2018). BRD4 is found at essentially all active promoters and a significant fraction of active enhancers in both normal and transformed cell types (Anand et al., 2013).

BET inhibitors (BETi) disrupt BET protein binding to acetylated lysine residues of chromatin and suppress the transcription of various genes, including oncogenes and oncogenic transcription factors. BETi is emerging as one of the most promising drugs to treat various types of cancer. There are several classes of BETi depending on whether they bind the BD of BET proteins noncovalently, bivalently, or if they also target BET proteins for degradation (Kulikowski et al., 2021). Noncovalent BETi has the largest number of currently available BETi, they can bind bromodomains of BET proteins noncovalently and compete with acetylated peptides, thus displacing BET proteins from acetylated chromatin (Filippakopoulos et al., 2010; Nicodeme et al., 2010). JQ1, IBET-762, IBET-151, OTX015, and ZEN-3694 all belong to this type, and they have shown antitumor activity in both cancer cell lines and murine cancer models (Dawson et al., 2011; Delmore et al., 2011; Boi et al., 2015; Baldan et al., 2019). 

Although it is still not clear how BET confers cancer-specific susceptibility, BETi is effective in reducing the transcription of several oncogenes (Delmore et al., 2011; Lovén et al., 2013; Fowler et al., 2014) and is potentially effective in treating various types of cancers including pancreatic ductal adenocarcinoma, leukemia, ovarian cancer (Yokoyama et al., 2016) and mature B-cell lymphoma (Dawson et al., 2011; Sahai et al., 2014; Boi et al., 2015; Mazur et al., 2015; Garcia et al., 2016). It is worth mentioning that besides cancer, BETi has also shown promising therapeutic benefits in cardiovascular (atherosclerosis (Tsujikawa et al., 2019) and heart failure (Anand et al., 2013; Duan et al., 2017)), autoimmune (juvenile idiopathic arthritis (Klein, 2018)) and metabolic diseases (obesity (Goupille et al., 2016; Duan *et al.*, 2020)). 

The molecular mechanisms of how BETis exert their anti-cancer function are not completely understood. Theoretically, inhibition of BRD4 would not only interfere with its oncogene targets but also other housekeeping genes essential for maintaining cell identity. It was hypothesized that BETi impacts the transcription of SE-associated genes more effectively than that of typical enhancers bound by BRD4 (Lovén et al., 2013). This “off-target” effect might be exacerbated with higher doses, which highlights the importance of discovering effective biomarkers to help visualize drug maximum activity and supervising dose control. 

Treatment-associated toxicity, drug resistance, and lack of predictive biomarkers have limited BETi’s progression in clinical trials (Sarnik et al., 2021). Future studies defining the mechanism of BETi activity, finding predictive biomarkers to predict sensitivity to BETi, and identifying potent combinational drugs would help prevent toxicities and facilitate its clinical success as anti-cancer agents (Shorstova et al., 2021).

*CDK7 inhibitors*

Cyclin-dependent kinase 7 (CDK7) drives cell cycle progression through the phosphorylation of cell cycle CDKs. CDK7 also phosphorylates RNA polymerase II which permits transcription at active genes. CDK7 is upregulated in various types of cancers including estrogen receptor-positive breast cancer (Patel et al., 2016), gastric cancer (Wang et al., 2016), triple-negative breast cancer(Li et al., 2017), ovarian cancer(Zhang et al., 2017) and oral squamous cell carcinoma(Jiang et al., 2019). CDK7 inhibitors are emerging as promising cancer therapeutic targets. Their anti-tumor effect is mediated through both cell cycle arrest and inhibition of oncogenic transcriptional programs. Examples of CDK7 inhibitors include covalent inhibitors such as THZ1 (Kwiatkowski et al., 2014), THZ2 (Zhang *et al.*, 2020), and SY-1365 (Hu et al., 2019) and noncovalent inhibitors such as BS-181 (Ali et al., 2009) and LDC4297 (Kelso et al., 2014).

CDK7 inhibition is most effective in suppressing SE-linked oncogenic transcription compared with other genes without SE association (Chipumuro et al., 2014; Eliades et al., 2018; Cao et al., 2019). SE is loaded with PolII, co-activators, Mediator complex, and transcription factors. And it is shown that SE-associated genes are particularly sensitive to small perturbations in CDK7 function and PolII-mediated transcription (Kwiatkowski et al., 2014). Treatments with covalent inhibitors inhibit downstream phophorylation of Pol II (Hu et al., 2019). CDK7 inhibitors also exert their anti-cancer function by reducing levels of SE-associated oncogenic TFs (Hu *et al.*, 2019). CDK7 inhibition leads to reduced recruitment of oncogenic TFs and the repression of associated oncogene expression (Yuan et al., 2022).

Due to its essential role in cell cycle progression, inhibition of CDK7 causes cell cycle arrest (Ali et al., 2009; Chipumuro et al., 2014; Kelso et al., 2014; Choi et al., 2019; Olson et al., 2019). The extent and timing of cell cycle arrest vary among different cancer types: LDC4297 causes G1 arrest in A549 lung cancer cells, but in HCT116 colon cancer cells only causes G2/M delay after extended incubation(Kelso *et al.*, 2014). 

### Genome editing to target enhancer

Another way to modify SE function in cancer is based on CRISPR/Cas9 gene editing system. The mutated form of transcription factor RUNX1 is associated with poorer outcomes in acute myeloid leukemia (AML). It is shown that CRISPR/Cas9 mediated knocking out of RUNX1 SE epicenter (a 24kb enhancer region inside the 170kb SE) results in repression of RUNX1 and higher apoptosis of AML cells (Mill et al., 2019). In a subset of T-cell acute lymphoblastic leukemia (T-ALL) cases, there are indels in a conserved noncoding region that create an SE upstream of the *TAL1* oncogene through introducing MYB transcription factor binding motifs. CRISPR/Cas9 experiments to cut out the mutated site resulted in the collapse of the SE and abrogation of *TAL1* expression (Mansour et al., 2014).

A few clinical trials have been completed or are ongoing that leverage NHEJ-mediated genetic disruption of *BCL11A* enhancer. Another way to modify enhancers for therapeutic purposes without introducing double-strand breaks is to base edit. CRISPR/Cas9 system and a cytidine deaminase enzyme could be fused together to mediate cytidine to uridine conversion and subsequently C to T substitution at the target site (Komor et al., 2016; Gehrke et al., 2018). A single therapeutic base edit of the *BCL11A* enhancer in patient-derived human hematopoietic stem and progenitor cells (HSPCs) prevents sickling and globin chain imbalance in their erythroid progeny (Zeng et al., 2020).

## Future directions

The function of enhancers in tumorigenesis has been the target of intensive research efforts for some years. It is foreseeable that more types of cancer would be found to be related to enhancer reprogramming. Identification of major enhancers, including SEs associated with different types of cancer and subgroups, would pave the way for finding more potential targets for treatment. Targeting both *cis* and *trans* factors in enhancer function has been utilized in cancer therapy through genome editing and anti-cancer agents (BETi and CDK7i), although the molecular mechanisms are not completely understood. There are issues associated with these agents’ progression in clinical trials. Defining mechanisms of activity and finding suitable biomarkers would aid their successful translation in cancer therapy. It is shown that enhancers also play important roles in cancer therapy resistance recently, and research on the molecular mechanism of enhancer function would enable more strategies to resolve therapy resistance.
